# Enhanced effect of X-rays in the presence of a static magnetic field within a 3D pancreatic cancer model

**DOI:** 10.1259/bjr.20220832

**Published:** 2023-01-14

**Authors:** Gabrielle Wishart, Priyanka Gupta, Andrew Nisbet, Eirini Velliou, Giuseppe Schettino

**Affiliations:** 1 Bioprocess and Biochemical Engineering Group (BioProChem), Department of Chemical and Process Engineering, University of Surrey, Guildford, UK; 2 Department of Physics, University of Surrey, Guildford, UK; 3 Department of Targeted Intervention, Centre for 3D Models of Health and Disease, Division of Surgery and Interventional Science, University College London (UCL), London, UK; 4 Department of Medical Physics and Biomedical Engineering, University College London (UCL), London, UK; 5 National Physical Laboratory, Teddington, UK

## Abstract

**Objective:**

To evaluate the impact of static magnetic field (SMF) presence on the radiation response of pancreatic cancer cells in polyurethane-based highly macro-porous scaffolds in hypoxic (1% O_2_) and normoxic (21% O_2_) conditions, towards understanding MR-guided radiotherapy, shedding light on the potential interaction phenomenon between SMF and radiation in a three-dimensional (3D) microenvironment.

**Methods:**

Pancreatic cancer cells (PANC-1, ASPC-1) were seeded into fibronectin-coated highly porous polyethene scaffolds for biomimicry and cultured for 4 weeks in *in vitro* normoxia (21% O_2_) followed by a 2-day exposure to either *in vitro* hypoxia (1% O_2_) or maintenance in *in vitro* normoxia (21% O_2_). The samples were then irradiated with 6 MV photons in the presence or absence of a 1.5 T field. Thereafter, *in situ* post-radiation monitoring (1 and 7 days post-irradiation treatment) took place via quantification of (i) live dead and (ii) apoptotic profiles.

**Results:**

We report: (i) pancreatic ductal adenocarcinoma hypoxia-associated radioprotection, in line with our previous findings, (ii) an enhanced effect of radiation in the presence of SMFin *in vitro* hypoxia (1% O_2_) for both short- (1 day) and long-term (7 days) post -radiation analysis and (iii) an enhanced effect of radiation in the presence of SMF in *in vitro* normoxia (21% O_2_) for long-term (7 days) post-radiation analysis within a 3D pancreatic cancer model

**Conclusion:**

With limited understanding of the potential interaction phenomenon between SMF and radiation, this 3D system allows combination evaluation for a cancer in which the role of radiotherapy is still evolving.

**Advances in knowledge:**

This study examined the use of a 3D model to investigate MR-guided radiotherapy in a hypoxic microenvironment, indicating that this could be a useful platform to further understanding of SMF influence on radiation.

## Background

Pancreatic ductal adenocarcinoma (PDAC) is a notoriously lethal disease with unique hallmarks that challenge current treatments.^1–3^ More specifically, the PDAC tumour microenvironment (TME) hosts a heterogeneous population of different cell types including pancreatic stellate cells, which once activated by cancer cells, secrete extracellular matrix (ECM) proteins in high volume, creating dense desmoplasia.^4–7^ This desmoplastic reaction, along with chaotic cancer cell growth causes the collapse of blood vessels, impairing chemotherapy delivery and creating large expanses of low oxygen (hypoxia) impairing radiotherapy efficiency.^8–12^ As a result of this complex TME pancreatic cancer is extremely treatment resistant. Moreover, non-specific symptoms resulting in late diagnosis and high metastatic occurrence elucidate devastatingly low 5 and 10 year survival rates of just 9 and 1%.^13^ These figures have failed to improve in line with other cancer survival rates in the last 50 years.^1,13,14^

The treatment of PDAC falls mainly to chemotherapy as only 8–20% of patients are eligible candidates for curative surgery at the time of diagnosis.^13^ Chemotherapeutic options for PDAC include Gemcitabine and Capecitabine as well as FOLFIRINOX.^15–18^ The American Society of Clinical Oncology suggest the consideration of radiotherapy for localised progression or stable disease after a 6 month period of chemotherapy.^19,20^ Moreover, Cancer Research UK (2020) states that radiotherapy is only utilised for 5% of PDAC patients. The utility of radiotherapy for pancreatic cancer is thought to be still evolving. This is due to conflicting clinical trials from Europe and America coupled with a lack of clinical adjuvant chemoradiotherapy data.^21–24^ The delivery of radiotherapy has advanced in recent years, with magnetic resonance (MR)-guided radiotherapy allowing for real-time imaging transforming radiation treatment planning for more precise dose delivery sparing organs at risk.

The use of MR-guided radiotherapy has prompted research into the potential impact of the static magnetic field (SMF) on human health and biological responses to radiation. The World Health Organisation (WHO) states that the data to suggest health risk effects of SMFs is insufficient.^25^ This was a result of reviewing biological effects of 0–14 T SMF *in vitro*, animal, human and epidemiology studies.^25^ Moreover, it is widely accepted that the use of MRI technologies are safe to apply in the clinic.^26,27^ However, few data exist studying the interaction between SMFs and radiation; therefore, it is of clinical relevance to analyse the potential interaction phenomena of ionising radiation and SMF combination on cancerous cells and cancerous cells in a TME at the level of exposure experienced in MR-guided radiotherapy. Reported changes to physiology as well as the yield and complexity of DNA damage by radiation in the presence of SMFs have been discussed and reviewed in literature.^28,29^ Similarly to the WHO findings, the amount of data to support radiation and SMF synergism are thought to be insufficient, with challenges in study comparison due to discrepancies in experimental set ups, radiation dosimetry and analysis.^29^ Despite this knowledge gap being present in literature, hypotheses exist around whether SMF may stimulate a biological response to radiation injury in a synergistic effect, including: (i) the modification of one or more steps in the DNA damage response, (ii) increasing the yield of lifetime of reactive oxygen species (ROS) (responsible for indirect DNA damage) or (iii) SMF influence of intercellular signalling impacted in non-targeted radiation-induced effects.^29^

As mentioned, only a few research articles exist reporting SMF in combination with radiation and this potential synergism, with the majority of these research studies utilising traditional 2D cell culture methods. For example, Feng et al.,^30^ evaluated the effects of 0.5 T SMF exposure 1 h post-radiation (10 Gy) to find an increase in apoptosis and a decreased clonogenic survival in human adenocarcinoma cells (A549).^30^ Moreover, Politanski *et al.,* (2013) also studied post- (up to 2 h) radiation (3 Gy) effects of 0.005 T SMF exposures to report increased levels of ROS in rat lymphocytes.^31^ Furthermore, Zhang et al.,^32^ studied the effects of 1 T SMF on 15 different cell lines to find that SMF does not impact cell cycle or cell death, however, at a higher density SMF reduced cell numbers in six out of seven solid human cancer cell lines.^32^ In contrast, Nath et al.,^33^ studied Chinese hamster lung cells (CCL16) to find no difference in clonogenic survival and recovery from sublethal damage of cells exposed to 2 T SMF during 30 MV X-rays up to 30 Gy in both hypoxic and normoxic conditions.^33^ Moreover, Wang et al.,^34^ utilised an MR-Linac (1.5 T SMF during 6 MV X-rays) to find that SMF exposure did not significantly impact survival of two human head and neck cancer and two lung cancer cell lines *in vitro*.^34^ Variations in SMF delivery protocols and measured biological endpoints facilitate difficulty in comparing studies that exist to investigate the potential effect of ionising radiation and SMF synergism. Moreover, the study of 2D cell culture techniques in vitro present challenges in reproducibility of in vivo properties.^35–40^ Unrealistic pre-clinical models impair treatment success at clinical level, thus the need to develop in vitro models that capture features of the TME are of clinical relevance when studying new modalities and their biological end points.^38^

Tissue engineering is facilitating the evolution of 3D models to recapitulate diverse ecosystems of TMEs for the application of treatment screening. More specifically, 3D models are more advanced than 2D cell culture systems supporting more realistic micro-architecture, cell-to-cell and cell-to-cell matrix interactions, spatial orientation and stiffness as well as allowing for the addition of ECM proteins, additional cells of the TME and environmental gradients.^35–40^^41^ 3D models have been reported in literature for radiation treatment screening for PDAC, these include *spheroid* and *polymeric* models. Spheroid models have been utilised to report radiation dose (0–6 Gy) dependent sensitivity,^42^ report co-cultured mediated radioresistance,^43^ and support new radiation modality testing (proton and boron neutron capture therapy).^44,45^ *Polymeric scaffolds* developed from biocompatible polymers allow finely tuned stiffness and internal architecture, more realistic spatial orientations and interactions of cells/cell matrix, as well as long-term cell culture.^35–40^ Previously, we have reported a highly macro-porous polyurethane (PU) polymeric scaffold supporting long term PDAC cell culture (35 days) and in vivo-like traits such as dense cellular masses, collagen-I secretion and environmental gradients.^36^ Furthermore, this model has also supported chemotherapy and radiotherapy screening up to 17 days post-treatment (which is the longest reported *in vitro* post-treatment timeframe),^37^ and hypoxia associated radioresistance up to 7 days post-radiation treatment.^39^

There are few studies that investigate the impact of SMF on 3D cell culture. Izzo et al.,^46^ developed a miniaturised optically accessible bioreactor facilitating the 3D culture of human neuroblastoma cells (SH-SY5Y) to find that SMF exposure did not influence cell metabolic activity.^46^ Furthermore, there are even less studies that investigate the impact of SMF in combination with radiation in 3D cell culture. Nicosia et al.,^47^ investigated PDAC organoids exposed to 1.5 T SMF and 6 Gy (7 MV flattening filter free photon beam together with a 1.5 T MR unit) to report combination treatment reduced cellular viability, increased apoptotic marker (Caspase 3/7) and reduced organoid size as compared to monotherapy.^47^

To the best of our knowledge, there are no polymeric 3D PDAC models to investigate SMF exposure in combination with radiation under hypoxia (an important radiation treatment limiting hallmark of PDAC). *Therefore, the aim of this work is to investigate the response of PDAC cells in an established long-term hypoxic 3D scaffold to SMF and SMF in combination with radiation*. Overall, we report for the first time: (i) PDAC hypoxia-associated radioprotection, in line with our previous findings,^39^ (ii) an enhanced effect of radiation in the presence of SMF in *in vitro* hypoxia (1% O_2_) for both short- (1 day) and long-term (7 days) post-radiation analysis and (iii) an enhanced effect of radiation in the presence of SMF in *in vitro* normoxia (21% O_2_) for long-term (7 days) post-radiation analysis within a 3D pancreatic cancer model.

## Methods

### Scaffold fabrication and surface modification

Polymeric scaffolds were fabricated via the thermally induced phase separation (TIPS) method as reported previously^36,48^ (Supplementary Figure 1). More specifically, 3 g of polyurethane (PU) beads (Noveon, Belgium) was dissolved in 60 ml dioxane (5% *w/v*) (99.8% anhydrous pure, Sigma-Aldrich, Merck, UK) for 48 h before the solution was quenched at –80°C for 3 h. The solvent was removed via freeze-drying in a polyethylene glycol (PEG) bath at –15°C under 0.01 mbar vacuum pressure for 72 h. Scaffolds were snap frozen in liquid nitrogen following immediate cutting into 5 × 5 × 5 mm^3^ cubes. Thereafter, the scaffolds were sterilised via 70% ethanol submersion (3 h) and UV ray exposure (1 h). The average pore size of the scaffold was 100–150 µm, the porosity was 85–90%, and the elastic modulus 20 ± 2 kPa, with stiffness similar to ex vivo high stiffness diseased PDAC tissue, as previously reported.^36,48–51^ Thereafter, the scaffolds were surface modified (adsorption) to enable coating with fibronectin (*i.e.* an ECM protein extensively present in the PDAC TME for ECM biomimicry). We have previously reported physiological behaviour of PDAC cells in the presence of fibronectin (dense cell aggregates, collagen-I secretion by the cancer cells, and realistic environmental gradients) compared to sparser cell organisation and no collagen production in uncoated scaffolds.^36^ Briefly, for surface modification with fibronectin, the scaffolds were centrifuged in phosphate buffered saline (PBS, Sigma-Aldrich, Merck, UK) for 10 min at 2500 rpm, then centrifuged in fibronectin solution (25 µg ml^–1^) for 20 min at 2000 rpm, before finally being centrifuged in PBS for 10 min at 1500 rpm.

Supplementary Figure 1.

### Cell culture

The 3D cell culture (in the scaffolds) was accomplished as described previously.^36^ More specifically, human pancreatic adenocarcinoma cells (PANC-1 and AsPC-1) (ATCC) were initially expanded in 2D flasks, in Dulbecco’s Modified Eagle’s Medium (DMEM) with high glucose (Sigma-Aldrich, Merck, UK) supplemented with 10% foetal bovine serum (Fisher Scientific, UK), 1% penicillin/streptomycin (Fisher Scientific, UK), and 2 mM L-glutamine (Sigma-Aldrich, Merck, UK) in 37°C with 21% O_2_ and 5% CO_2_. PDAC cells were passaged when 80–90% confluency was reached, until the appropriate cell number for the 3D experiments was obtained. Thereafter, 0.5 × 10^6^ PDAC cells were seeded per scaffold (re-suspended in 30 µL of cell culture media) and placed in 24-well plates. Thereafter, the scaffolds were placed in an incubator for 1 h to ensure adherence. Therein, 1.5 ml of cell culture media was added to each well, this was replaced every 2 days and each 24-well plate was replaced after 1 week to avoid cell egress from scaffolds and cell confluency on the bottom of each well. Incubation of all scaffolds took place in a humidified incubator at 37°C with 21% O_2_ and 5% CO_2_ (in vitro normoxia) for 28 days (4 weeks). Thereafter, half of the scaffolds were moved to in vitro hypoxic conditions at 37°C with 1% O_2_ and 5% CO_2_ in a Ruskinn InvivO2 300 workstation (Baker Ruskinn, Ltd., Bridgend, UK) for a 2 day pre-treatment exposure to low oxygen. Post-treatment analysis took place at 1 and 7 days post-treatment in both: (i) in vitro normoxic (21% O_2_) and (ii) in vitro hypoxic (1% O_2_) culture conditions.

### Radiation treatment and static magnetic field exposure

Radiation treatments were performed with using 6 MV photons from a Linear Accelerator (Synergy model from Elekta, Sweden). The Linac is located in a bunker with a variable electromagnet (GMW, US, 250 mm electromagnet, model: 3474–140), with a maximum field strength of 2.2 T. The magnet can be positioned so that the Linac isocentre coincide with the centre of the magnet. The dosimetry was performed according to the IPEM 1990 Code of Practise^52^ using alanine as a transfer standard^53^ to take into account the effect of the magnetic field. The magnet pole diameter was 250 mm and the gap between the poles was set to 70 mm which allowed for a 1.5 T magnetic field with a current of 139 A. The strength of the magnetic field was checked using a Transverse Hall magnetic probe. The photon beam emerging from the Linac was shaped to deliver a field size of 5.89 × 13.64 cm at the centre of the magnet poles avoiding radiation scattering from the poles but covering the whole samples.

In order to expose 3D scaffolds to radiation and in combination with static magnetic field at the National Physical Laboratory, a 3D printed scaffold holder (polypropylene filament with a density of 0.89 g/cm^3^) in the shape of a flask (44 × 27×150 mm) and phantom/flask holder (RS PRO Brand Black PLA filament) (30.5 × 50.5×115 mm) (Supplementary Figure 1) were printed at the University of Surrey using a Ultimaker S3 3D printer (Ultimaker, Amsterdam).

Control scaffolds were used to account for cellular stress during transportation from the University of Surrey to the National Physical Laboratory. More specifically, control scaffolds were transported to the National Physical Laboratory alongside treatment samples and placed in the phantom/flask holder for the radiation time period without any radiation dose applied. Thereafter, hypoxic scaffolds were placed back in 1% O_2_, normoxic scaffolds were placed back at 21% O_2_, and all scaffolds were analysed after 1 and 7 days post-radiation treatment. Overall, all conditions included; (i) control (no radiation, no SMF), (ii) radiation treatment (6 Gy) (iii) static magnetic field exposure (1.5 T SMF); and (iiii) radiation treatment (6 Gy) and SMF (1.5 T) exposure combined. All conditions were repeated for both 21% O_2_ culture and 1% O_2_ culture.

### Live/Dead analysis via confocal imaging

The distribution of live and dead cells within scaffolds for all conditions under study (treated and untreated for both normoxic and hypoxic scaffolds) was evaluated via the Live/Dead Viability/Cytotoxicity Kit (Molecular Probes, Thermo Scientific, UK). Calcein-AM is a fluorogenic esterase substrate that is hydrolysed to label cells that are esterase activity positive with an intact membrane (retaining esterase products) with a green-fluorescent product calcein. Ethidium Homodimer is a red-fluorescent nucleic acid stain that targets cells with compromised membranes only.

Scaffolds were snap frozen at time points of 1 and 7 days post-treatment in liquid nitrogen for 20 min and preserved thereafter at –80°C as previously described.^36,37,39^ Thereafter, the scaffolds were sectioned and stained with 2 µm of Calcein-AM (4 mM stock) and 4 µm of Ethidium Homodimer (2 mM stock) and incubated at 37°C for 1 h. Thereafter, the samples were washed twice with PBS and visualised using a Nikon Ti-Eclipse inverted confocal microscope (Nikon Instruments, Europe).

### Caspase 3/7 analysis via confocal imaging

The distribution of apoptotic cells within scaffolds for all conditions under study was evaluated via Caspase 3/7 analysis. More specifically, scaffolds were snap frozen at time of points 1 and 7 days post-treatment in liquid nitrogen for 20 min and preserved thereafter at –80°C. The scaffolds were sectioned and stained with Cell Event Caspase 3/7 green detection reagent (Fisher Scientific, UK) and DAPI (Fisher Scientific, UK) for 1 h at 37°C. Thereafter, the samples were washed with PBS and visualised using a Nikon Ti-Eclipse inverted confocal microscope (Nikon Instruments, Europe).

### Confocal laser scanning microscopy

Live/Dead, Caspase 3/7 analysis were imaged on a Nikon Ti-Eclipse inverted confocal microscope (Nikon Instruments, Europe) and processed with the NIS-Elements software, using 405, 488, and 647 lasers for DAPI (blue), Calcein AM and Caspase 3/7 (green), and Ethidium Homodimer staining, respectively. Images were captured at a 10× objective and 10 µm Z-stack distance. Multiple scaffolds (*n* = 3), scaffold sections (*n* = 3), and scaffold areas (*n* = 2) were imaged for each condition under study to ensure reproducibility. The images presented here are representative images from each condition.

### Image analysis

ImageJ^®^ software (Wayne Rasband, NIH, Bethesda, MD) was utilised to quantify spatial characterisation of: (i) live areas *vs* dead areas and (ii) Caspase 3/7 positive areas *vs* DAPI. Multiple scaffolds (*n* = 3), scaffold sections (*n* = 3), and scaffold areas (*n* = 2) were analysed per condition to ensure reproducibility of results. The bars in each bar graph of the results represent averages of percentage areas of each fluorescence channel.

### Statistical analysis and data analysis

Graph Pad Prism^®^ was utilised to determine statistical significance (*p* < 0.05). Analysis of variance (ANOVA), followed by Tukey’s multiple comparison test were employed. Standard error of the mean was used to determine error bars in the bar graphs. Where data were normalised with respect to the control, the following equation was employed: % Caspase 3/7 area = ((treatment positive area)/(control positive area)) ×100.

## Results

The evaluation of cell viability and cell apoptosis in 3D scaffolds were monitored for 1 and 7 days post- radiation with live/dead and Caspase 3/7 staining for PDAC cell lines PANC-1 (Figures 1–4) and AsPC-1 (Figures 5–8).

**Figure 1.**
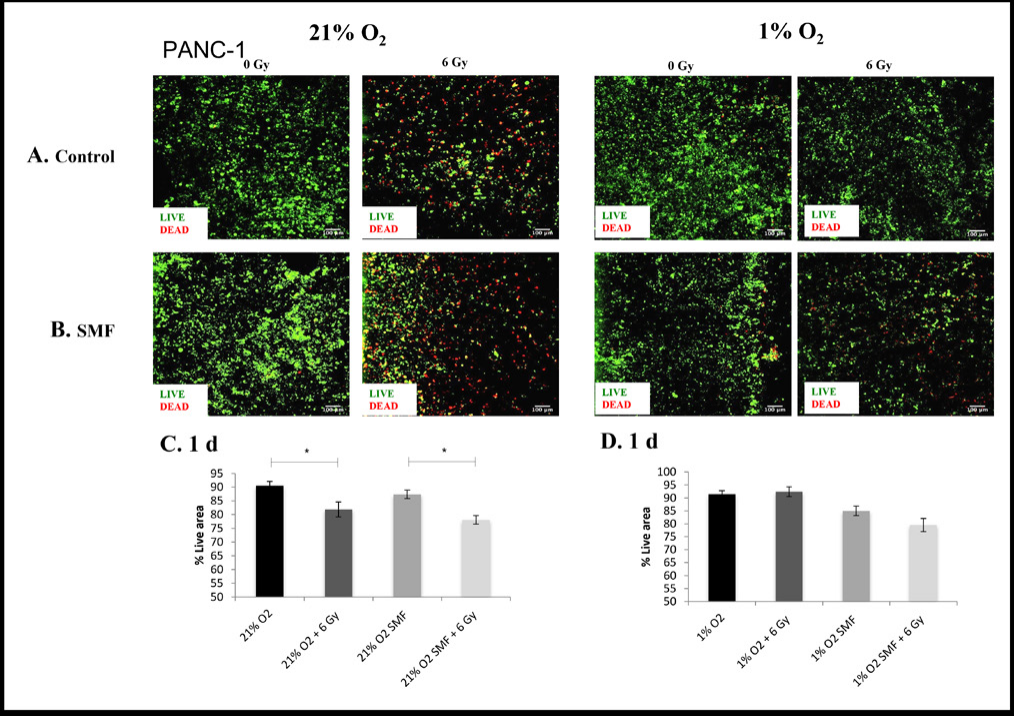
PANC-1 cell viability (live/dead staining) following radiation treatment (6 Gy) in combination with SMF (1.5 T) exposure in 3D scaffolds for 21% O_2_ and 1% O_2_ 1 day post-treatment: (**A, B**) Representative images of scaffold sections for live (green)/dead (red) staining, 1 day post-treatment (**C, D**). Equivalent image analysis-based quantification of the percentage of live (green) image areas for A and B. Multiple scaffolds (≥3), scaffold sections (≥3) and images were analysed, mean values are presented. (* = p < 0.05). SMF, static magnetic field.

**Figure 2.**
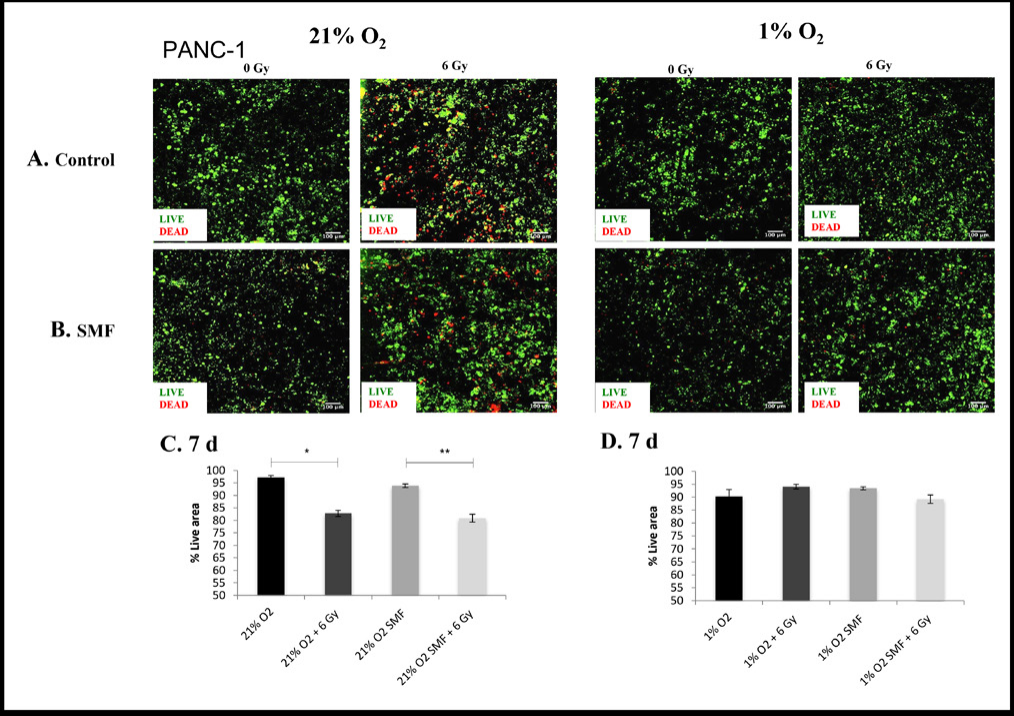
PANC-1 cell viability (live/dead staining) following radiation treatment (6 Gy) in combination with SMF (1.5 T) exposure in 3D scaffolds for 21% O_2_ and 1% O_2_ 7 days post-treatment: (**A, B**) Representative images of scaffold sections for live (green)/dead (red) staining, 7 day post-treatment (**C, D**). Equivalent image analysis based quantification of the percentage of live (green) image areas for A and B. Multiple scaffolds (≥3), scaffold sections (≥3) and images were analysed, mean values are presented. (** = p < 0.01) (* = p < 0.05). SMF, static magnetic field.

**Figure 3.**
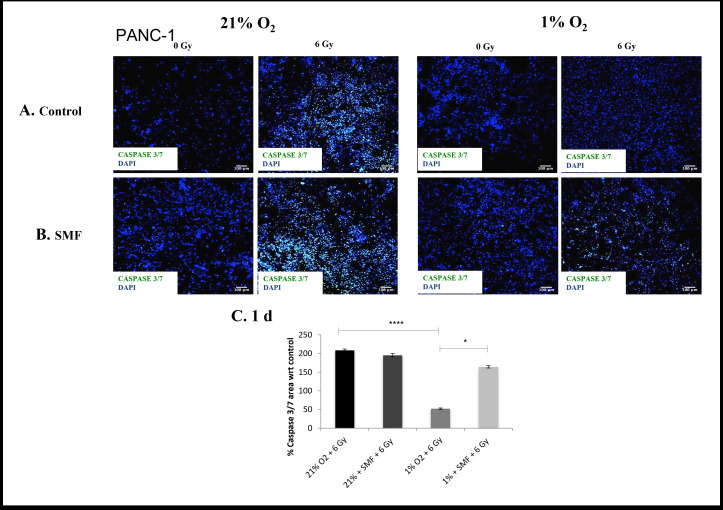
PANC-1 apoptotic assay (Caspase 3/7 staining) following radiation treatment (6 Gy) in combination with SMF (1.5 T) exposure in 3D scaffolds for 21% O_2_ and 1% O_2_ 1 day post-treatment: (**A, B**) Representative images of scaffold sections for Caspase 3/7 (green) and DAPI (blue) staining, 1 day post-treatment (**C**). Equivalent image analysis based quantification of the percentage of live (green) image areas for A and B. Multiple scaffolds (≥3), scaffold sections (≥3) and images were analysed, mean values are presented. (*** = p < 0.001) (* = p < 0.05). SMF, static magnetic field.

**Figure 4.**
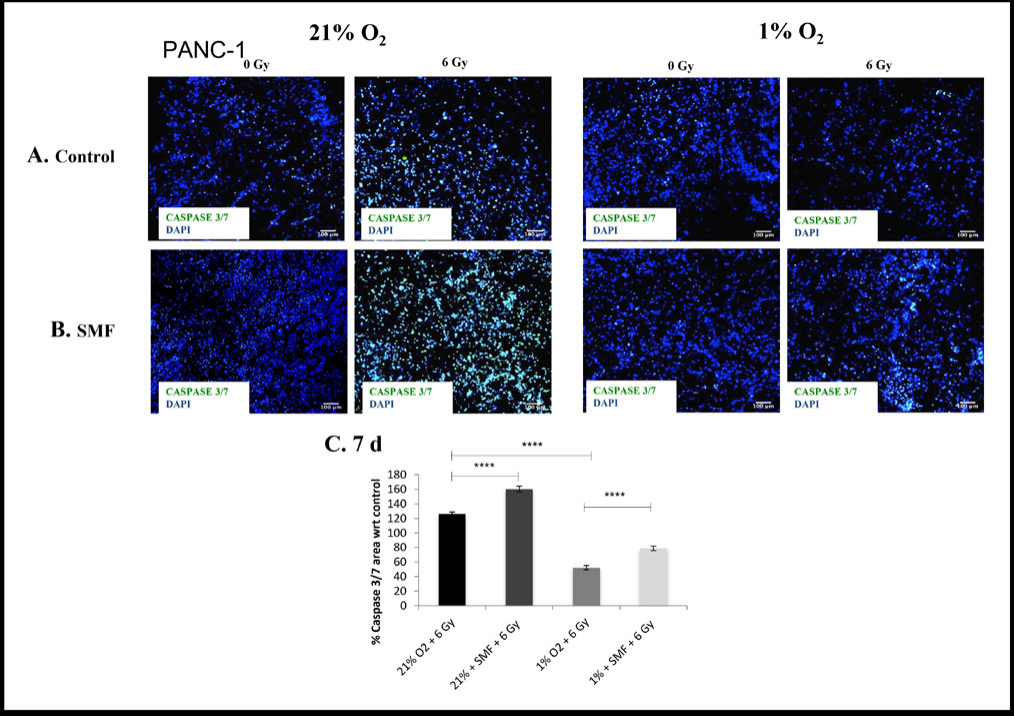
PANC-1 apoptotic assay (Caspase 3/7 staining) following radiation treatment (6 Gy) in combination with SMF (1.5 T) exposure in 3D scaffolds for 21% O_2_ and 1% O_2_ 7 days post-treatment: (**A, B**) Representative images of scaffold sections for Caspase 3/7 (green) and DAPI (blue) staining, 7 day post-treatment (**C**). Equivalent image analysis based quantification of the percentage of live (green) image areas for A and B. Multiple scaffolds (≥3), scaffold sections (≥3) and images were analysed, mean values are presented. (**** = p < 0.0001). SMF, static magnetic field.

**Figure 5.**
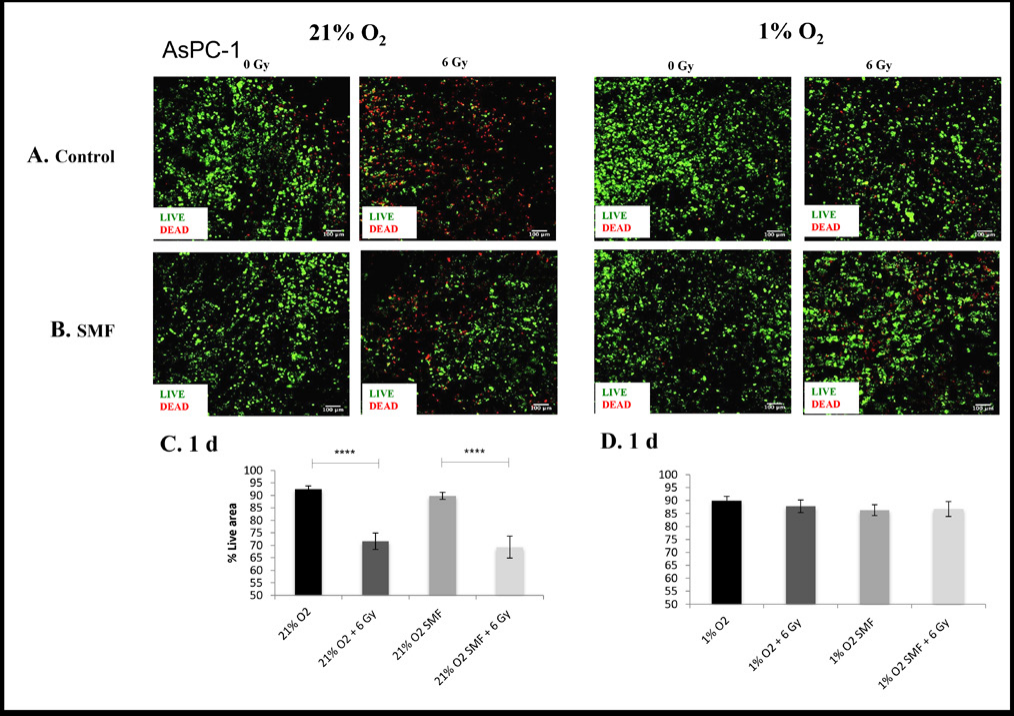
AsPC-1 cell viability (live/dead staining) following radiation treatment (6 Gy) in combination with SMF (1.5 T) exposure in 3D scaffolds for 21% O_2_ and 1% O_2_ 1 day post-treatment: (**A, B**) Representative images of scaffold sections for live (green)/dead (red) staining, 1 day post-treatment (**C, D**). Equivalent image analysis based quantification of the percentage of live (green) image areas for A and B. Multiple scaffolds (≥3), scaffold sections (≥3) and images were analysed, mean values are presented. (**** = p < 0.0001). SMF, static magnetic field.

**Figure 6.**
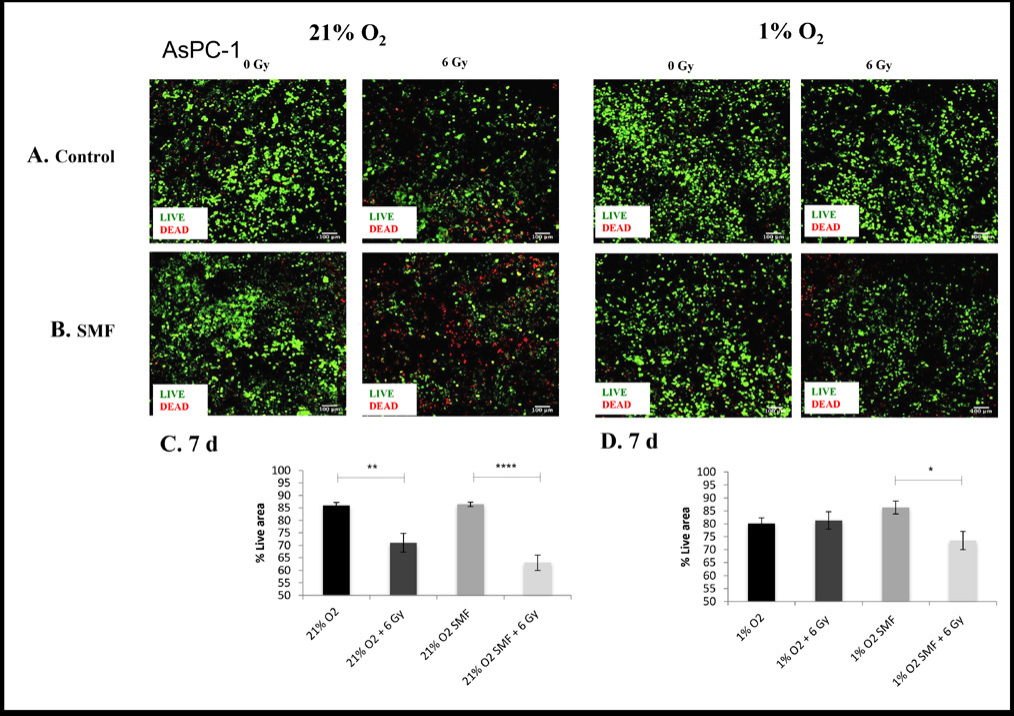
AsPC-1 cell viability (live/dead staining) following radiation treatment (6 Gy) in combination with SMF (1.5 T) exposure in 3D scaffolds for 21% O_2_ and 1% O_2_ 7 days post-treatment: (**A, B**) Representative images of scaffold sections for live (green)/dead (red) staining, 7 day post-treatment (**C, D**). Equivalent image analysis based quantification of the percentage of live (green) image areas for A and B. Multiple scaffolds (≥3), scaffold sections (≥3) and images were analysed, mean values are presented. (**** = p < 0.0001) (** = p < 0.01) (* = p < 0.05). SMF, static magnetic field.

**Figure 7.**
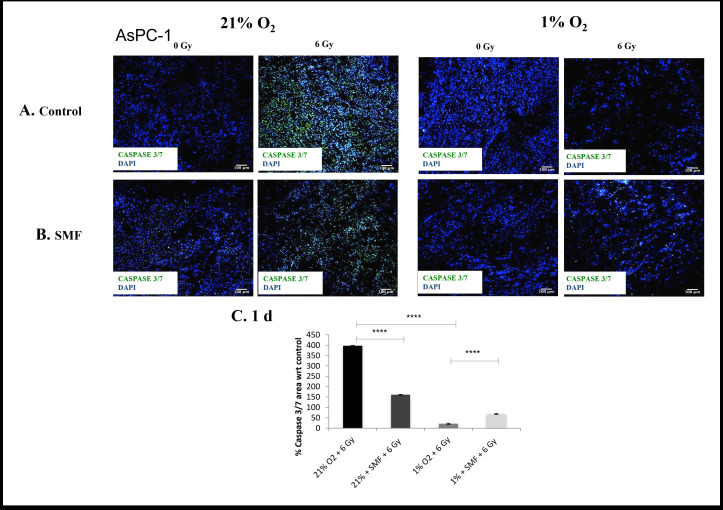
AsPC-1 apoptotic assay (Caspase 3/7 staining) following radiation treatment (6 Gy) in combination with SMF (1.5 T) exposure in 3D scaffolds for 21% O_2_ and 1% O_2_ 1 day post-treatment: (**A, B**) Representative images of scaffold sections for Caspase 3/7 (green) and DAPI (blue) staining, 1 day post-treatment (**C**). Equivalent image analysis based quantification of the percentage of live (green) image areas for A and B. Multiple scaffolds (≥3), scaffold sections (≥3) and images were analysed, mean values are presented. (**** = p < 0.0001). SMF, static magnetic field.

**Figure 8.**
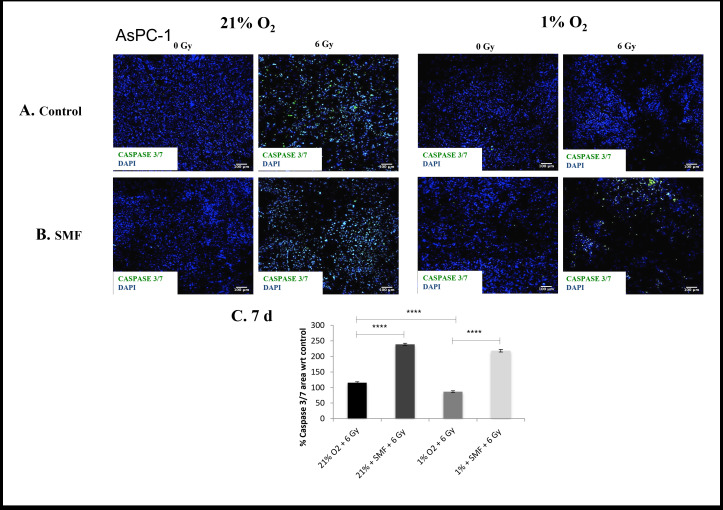
AsPC-1 apoptotic assay (Caspase 3/7 staining) following radiation treatment (6 Gy) in combination with SMF (1.5 T) exposure in 3D scaffolds for 21% O_2_ and 1% O_2_ 7 days post-treatment: (**A, B**) Representative images of scaffold sections for Caspase 3/7 (green) and DAPI (blue) staining 7 day post-treatment (**C**). Equivalent image analysis based quantification of the percentage of live (green) image areas for A and B. Multiple scaffolds (≥3), scaffold sections (≥3) and images were analysed, mean values are presented. (**** = p < 0.0001). SMF, static magnetic field.

Figures 1 and 2 summarise representative confocal images of scaffold sections showing the spatial distribution of Live/dead cell areas for 1 and 7 days post-radiation screening, as well as quantification of the percentage of total live and apoptotic areas in multiple images from multiple sections of multiple scaffolds of PANC-1 cells. The *p* values of this live cell area analysis are presented in Table 1. The results show a systematic trend of hypoxia-associated radioprotection in PANC-1 cells in 3D scaffolds. More specifically, we see a significant decrease in live cell area when 3D scaffolds are treated with radiation in *in vitro* normoxia, whereas we report no significant decrease in live cell area when 3D scaffolds are treated with radiation in *in vitro* hypoxia. This is in line with previous findings of hypoxia-associated radioprotection.^39^

**Table 1.** Statistical analysis evaluation of cell viability in 3D scaffolds for 1 and 7 days post-radiation for PANC-1 cells

	21%	21% + 6 Gy	21% + 1.5 **T**	21% + 1.5 **T** + 6 Gy	1%	1% + 6 Gy	1% + 1.5 **T**	1% + 1.5 **T** + 6 Gy
**1** day	90.5% (Std. error Mean: 1.6)	82% (Std. error Mean: 2.7)	87.4% (Std. error Mean: 1.6)	78% (Std. error Mean: 1.5)	91% (Std. error Mean: 1.3)	92% (Std. error Mean: 1.9)	84.9% (Std. error Mean: 1.8)	78% (Std. error Mean: 2.6)
*p-* value	*p* < 0.05		*p* < 0.05		ns		ns	

**7 days**	97% (Std. error Mean: 0.7)	83% (Std. error Mean: 1.2)	94% (Std. error Mean: 0.7)	81% (Std. error Mean: 1.6)	90% (Std. error Mean: 2.7)	94% (Std. error Mean: 0.9)	94% (Std. error Mean: 0.6)	89% (Std. error Mean: 1.6)
*p-* value	*p* < 0.05		*p* < 0.05		ns		ns	

To complement the cell viability trend, a measure of significant damage to the PANC-1 cells was recorded in the form of apoptotic cell presence for 1 and 7 days post-radiation analysis (Caspase 3/7 marker for apoptosis). Figures 3 and 4 summarise representative confocal images of scaffold sections showing the spatial distribution of Caspase 3/7 positive areas for 1 and 7 days post-radiation screening, as well as quantification of the percentage of total Caspase 3/7 area with respect to their relative controls in multiple images from multiple sections of multiple scaffolds of PANC-1 cells. The *p* values of this Caspase 3/7 analysis are presented in Table 2. The results show a systematic trend of an enhanced effect of radiation in the presence of SMF in *in vitro* hypoxia. More specifically, a significantly higher level of Caspase 3/7 was reported in *in vitro* hypoxia (1% O_2_) treated with radiation (6 Gy) in combination with SMF (1.5 T) as compared to *in vitro* hypoxia (1% O_2_) treated with radiation (6 Gy) alone at both 1 and 7 days post-radiation analysis. Moreover, we also report this trend at 7 days post-radiation analysis in *in vitro* normoxia.

**Table 2.** Statistical analysis evaluation of apoptotic marker presence in 3D scaffolds for 1 and 7 days post- radiation for PANC-1 cells

	21% + 6 Gy	21% + 1.5 **T** + 6 Gy	1% + 6 Gy	1% + 1.5 **T** + 6 Gy
**1** day	208.6% (Std. error Mean: 3.3)	195% (Std. error Mean: 4.4)	52.3% (Std. error Mean: 1.9)	164% (Std. error Mean: 3.2)
*p-* value	*p* < 0.001		*p* < 0.05	
**7 days**	125.8% (Std. error Mean: 3)	160.4% (Std. error Mean: 3.9)	52.2% (Std. error Mean: 3)	78.8% (Std. error Mean: 3.1)
*p*-value	*p* < 0.0001		*p* < 0.0001	

To ensure validity and reproducibility, this research was repeated with a more differentiated PDAC cell line (AsPC-1) (Figures 5–8). Overall, these data show consistent findings to the PANC-1 results despite the different level of differentiation. Figures 5 and 6 summarise representative confocal images of scaffold sections showing the spatial distribution of live/dead cell areas for 1 and 7 days post-radiation screening, as well as quantification of the percentage of total live and apoptotic areas in multiple images from multiple sections of multiple scaffolds of AsPC-1 cells. The *p* values of this live cell area analysis are presented in Table 3. The results show a systematic trend of hypoxia-associated radioprotection in AsPC-1 cells in 3D scaffolds. More specifically, we see a significant decrease in live cell area when 3D scaffolds are treated with radiation in *in vitro* hypoxia, whereas we report no significant decrease in live cell area when 3D scaffolds are treated with radiation in *in vitro* hypoxia. This is in line with previous findings of hypoxia-associated radioprotection.

**Table 3.** Statistical analysis evaluation of cell viability in 3D scaffolds for 1 and 7 days post-radiation for AsPC-1 cells

	21%	21% + 6 Gy	21% + 1.5 **T**	21% + 1.5 T + 6 Gy	1%	1% + 6 Gy	1% + 1.5 **T**	1% + 1.5 **T** + 6 Gy
**1** day	92.5% (Std. error Mean: 1.3)	72% (Std. error Mean: 3.3)	90% (Std. error Mean: 1.4)	69% (Std. error Mean: 4.4)	90% (Std. error Mean: 1.7)	88% (Std. error Mean: 2.5)	86% (Std. error Mean: 2.1)	87% (Std. error Mean: 2.8)
*p-* value	*p* < 0.0001		*p* < 0.0001		ns		ns	

**7 days**	86% (Std. error Mean: 1.2)	71% (Std. error Mean: 3.8)	86% (Std. error Mean: 0.9)	63% (Std. error Mean: 3)	80% (Std. error Mean: 2.2)	81% (Std. error Mean: 3.4)	86% (Std. error Mean: 2.5)	73% (Std. error Mean: 3.5)
*p-* value	*p* < 0.01		*p* < 0.0001		ns		*p* < 0.01	

Similarly to the evaluation of PANC-1 cells apoptotic cell presence for 1 and 7 day post-radiation were analysed for AsPC-1 cells. Figures 7 and 8 summarise representative confocal images of scaffold sections showing the spatial distribution of Caspase 3/7 positive areas for 1 and 7 days post-radiation screening, as well as quantification of the percentage of total Caspase 3/7 area with respect to their relative controls in multiple images from multiple sections of multiple scaffolds of AsPC-1 cells. The *p* values of this Caspase 3/7 analysis are presented in Table 4. The results show a systematic trend of an enhanced effect of radiation in the presence of SMF in *in vitro* hypoxia. More specifically, a significantly higher level of Caspase 3/7 was reported in *in vitro* hypoxia treated with radiation in combination with SMF as compared to *in vitro* hypoxia treated with radiation alone at both 1 and 7 days post-radiation analysis. Moreover, we also report this trend at 7 days post-radiation analysis in *in vitro* normoxia.

**Table 4.** Statistical analysis evaluation of apoptotic marker presence in 3D scaffolds for 1 and 7 days post- radiation for AsPC-1 cells

	21% + 6 Gy	21% + 1.5 **T** + 6 Gy	1% + 6 Gy	1% + 1.5 **T** + 6 Gy
**1** day	397.4% (Std. error Mean: 2.6)	161.2% (Std. error Mean: 1.9)	21% (Std. error Mean: 2.6)	68.9% (Std. error Mean: 2.4)
*p*-value	*p* < 0.001		*p* < 0.05	
**7 days**	115.7% (Std. error Mean: 2.6)	238.9% (Std. error Mean: 3.7)	86.7% (Std. error Mean: 2.6)	218.4% (Std. error Mean: 4.2)
*p*-value	*p* < 0.0001		*p* < 0.0001	

## Discussion

In this work, we report the impact of the SMF presence in combination with radiation treatment in *in vitro* hypoxia and *in vitro* normoxia in our recently developed polyurethane based highly macro-porous 3D scaffold. This system supports long-term (37 days) culture of PDAC cells with cell proliferation and distribution similar to that of reported mouse models for this time-frame.^36,54^ Here, PDAC cells were seeded in polymeric scaffolds and cultured for 4 weeks in *in vitro* normoxia (21% O_2_) followed by 2 days exposure to *in vitro* hypoxia (1% O_2_) or maintenance in *in vitro* normoxia. Thereafter, radiation treatment (6 Gy) and SMF (1.5 T) exposure followed by *in situ* post-treatment monitoring (1 and 7 days) (for short- and longer-term assessment) took place via quantification of live/dead and apoptotic profiles (Caspase 3/7). For radiation treatment, a dose of 6 Gy was selected based on our previously published radiation screening experiments and SMF exposure of 1.5 T was based on literature and clinical MR usage.^37^ The selection of oxygen percentage profiles was in line with literature as most papers report 0.1 to 10% O_2_ for hypoxic research^55^ ; moreover, we have previously tested 5% O_2_ to find hypoxic induced radioprotection,^39^ and selected a lower oxygen percentage (1% O_2_) to more accurately recapitulate PDAC TME partial pressures and physoxia reported in literature.^56^ PANC-1 and AsPC-1 cells lines were chosen as two PDAC cell lines of varying origin and differentiation, *i.e.* PANC-1 cells are poorly differentiated and AsPC-1 cells are moderately to highly differentiated.^57^ Cancer stem cells are generally regarding as more radioresistant than their differentiated equivalent due a high DNA repair capability, low level of ROS and slow proliferation (REF).^58^ We have observed similar response in both of the cell lines in this study, which is likely due to the fact that neither of them are stem cell lines although they exhibit different differentiation status.

This research reports: (i) PDAC hypoxia-associated radioprotection, *i.e.* increased cell viability profiles (Figures 1, 3, 5 and 7) and decreased cell apoptosis (Figures 2, 4, 6 and 8) for both short term (1 day post-radiation) and long-term (7 day post-radiation) analysis, inline with our previous findings of radioprotection in *in vitro* hypoxia.^39^ (ii) An enhanced effect of radiation in the presence of SMF in *in vitro* hypoxia (1% O_2_) for both short- (1 day) and long-term (7 days) (Figures 2, 4, 6 and 8) post-radiation analysis, *i.e.* increased apoptosis profiles in radiation treatment combined with SMF as compared to radiation treatment alone in *in vitro* hypoxia for PANC-1 and AsPC-1 cells. As well as (iii) a prolonged enhancement of radiation effect in the presence of SMF in *in vitro* normoxia (21% O_2_) post-radiation analysis for both cell lines (Figures 4 and 8), *i.e.* increased apoptosis profiles in radiation treatment combined with SMF as compared to radiation treatment alone in *in vitro* normoxia at 7 days post-treatment analysis.

To the best of our knowledge, this research is the first study to exploit a 3D highly porous polymeric scaffold for radiation response studies in combination with SMF in both normoxic and hypoxic cultures. Research into the combined effect of SMF and radiation has taken place in 2D and in various cell lines. As previously mentioned, Nath et al.,^33^ studied Chinese hamster lung cells (CCL16) to find no difference in clonogenic survival and recovery from sublethal damage of cells exposed to 2 T during 30 MV X-rays up to 30 Gy in both hypoxic and normoxic conditions.^33^ Wang et al.,^34^ utilised an MR-Linac (1.5 T SMF, 6 MV X-rays) to find that SMF did not significantly impact survival of two human head and neck cancer and two lung cancer cell lines *in vitro*.^34^ More recently, Yudhistiara et al.,^27^ reported that 1.0 T did not affect the clonogenic survival fraction of normal human lymphoblastoid cell line (TK6) during 1–4 Gy 6 MV photons.^27^ On the contrary, Feng et al.,^30^ evaluated the effects of 0.5 T SMF exposure 1 h post-radiation (10 Gy) to find an increase in apoptosis and a decreased clonogenic survival in human adenocarcinoma cells (A549).^30^ Moreover, Politanski *et al.,* (2013) also studied post (up to 2 h) radiation (3 Gy) effects of 0.005 T SMF exposures to report increased levels of ROS in rat lymphocytes.^31^ Furthermore, Zhang et al.,^32^ studied the effects of 1 T SMF on 15 different cell lines to find that SMF does not impact cell cycle of cell death, however, at a higher density SMF reduced cell numbers in six out of seven solid human cancer cell lines.^32^

As previously mentioned, there are a limited number of 3D PDAC models to investigate radiation treatments in combination with SMF. However, Nicosia *et al.* (2020) developed PDAC patient sample organoids to investigate the exposure of SMF and radiation combinations. Similar to our work, this research measured cellular viability and the apoptotic marker Caspase 3/7 to find that combination treatments (1.5 T SMF MR unit and 6 Gy. 7 MV flattening filter free photon beam) reduced cell viability and increased apoptosis as compared monotherapy as well as reduced organoid size.^47^ To the best of our knowledge this is the only other 3D model for PDAC to report a combined effect of SMF and radiation.

The application of MRI-guided radiotherapy has transformed radiation treatment planning for more specific dose delivery. However, few data exist studying the interaction between radiation and SMFs, therefore it is of clinical relevance to analyse the potential interaction phenomena of ionising radiation and SMF combination on cancerous cells and cancerous cells in a TME. Recent years have highlighted the importance of the TME and its effect on tumour prognosis and treatment resistance. The ability to recapitulate a complex TME ecosystem in a 3D model and identify treatment-screening profiles of new modalities such as MRI-guided radiotherapy is required to improve our understanding. At present, there is a significant gap in literature investigating the use of 3D models for long-term monitoring of the potential biological response influence of SMF in combination with radiation. Literature is attempting to understand and explain potential phenomena of radiation and SMF synergism. More specifically, Mohajer et al.,^29^ suggest the following hypotheses to be considered for the rationale behind potential synergism of radiation and SMF; (i) the modification of one or more steps in the DNA damage response, (ii) increasing the yield of lifetime of ROS (responsible for indirect DNA damage) or (iii) SMF influence of intercellular signalling impacted in non-targeted radiation-induced effects.^29^ Exposure of SMFs has previously been reported to protect cells from apoptosis via increases in ROS.^59^ It could be suggested that is similar to the effect we are reporting here, however further investigation is required to pinpoint the DNA damage effect of SMF and radiation combinations and assess a longer-term effects. The use of the live/dead assay identifies green fluorescence cells based on intact membranes and esterase activity and some cells selected may have received significant DNA damage, which would manifest at a later stage into a lack of colony formation. This may explain why, in this research, the apoptosis assay displays a more prominent effect of death after treatments as compared to the live/dead analysis. A typical method of assessing radiation- induced damage via effectively establishing reproductive integrity and therefore cell death in the form of colony presence or absence is the clonogenic assay. This analysis is not possible here due to the nature of the 3D scaffold and the inability to extract cells from the 3D model. This highlights the need to follow up this research with molecular analysis of DNA damage assessment. Furthermore, as 3D models with complex TME biomimicry emerge, there is a need for a universally accepted protocol to assess radiation-induced DNA damage *in situ*.^38^

## Conclusions

This work performed *in vitro* hypoxic radiation screening in combination with static magnetic field in our previously established scaffold-based PDAC model. PANC-1 and AsPC-1 scaffolds were cultured for 4 weeks in *in vitro* normoxia (21% O_2_) and then exposed to *in vitro* hypoxia (1% O_2_) (2 days) followed by radiation treatment (6 Gy) and SMF (1.5 T) exposure and *in situ* post-treatment monitoring (1 and 7 days) via quantification of: (i) cell viability (Live/dead) and (ii) apoptotic (Caspase 3/7) profiles. Our analysis revealed: (i) PDAC hypoxia-associated radioprotection, *i.e.* increased cell viability profiles and decreased cell apoptosis for both short- (1 day post- radiation) and long-term (7 day post-radiation) analysis, inline with our previous findings of radioprotection in *in vitro* hypoxia,^39^ , (ii) an enhanced effect of radiation in the presence of SMF in *in vitro* hypoxia (1% O_2_) for both short- (1 day) and long-term (7 days) post-radiation analysis and (iii) an enhanced effect of radiation in the presence of SMF in *in vitro* normoxia (21% O_2_) for long-term (7 days) post-radiation analysis for two PDAC cell lines within a 3D pancreatic cancer model.

To the best of our knowledge, this is the first study to report SMF and radiation combination treatment in a long term, 3D hypoxic polymeric scaffold model for PDAC. There are very limited studies investigating radiation and SMF combinations in 3D models and currently this is the first scaffold based 3D model investigating this potential interaction phenomenon for PDAC. Our system provides a platform for animal-free assessment of developing radiation modalities screening, allowing more advanced spatial patterns and long-term culture as compared to *in vitro* 2D culture. This work attempts to widen our knowledge of magnetic-enhanced radiotherapy, however, it is evident that further research into this area is required. Our future work hopes to address the response of PDAC in this system to other modalities such as proton beams. Moreover, our recently developed complex multicellular scaffold will be evaluated for treatment response studies under hypoxia to further develop understand the impact of the PDAC TME on radiation response.
