# Supplementary material for: Enhanced effect of X-rays in the presence of a static magnetic field within a 3D pancreatic cancer model
Source: Br J Radiol. 2023 Jan 14;96(1143):20220832. doi: 10.1259/bjr.20220832 (PMC9975369; doi:10.1259/bjr.20220832)
Supplement: Supplementary Figure 1. [file bjr.20220832.suppl-01.docx]

**Appendix A**

**
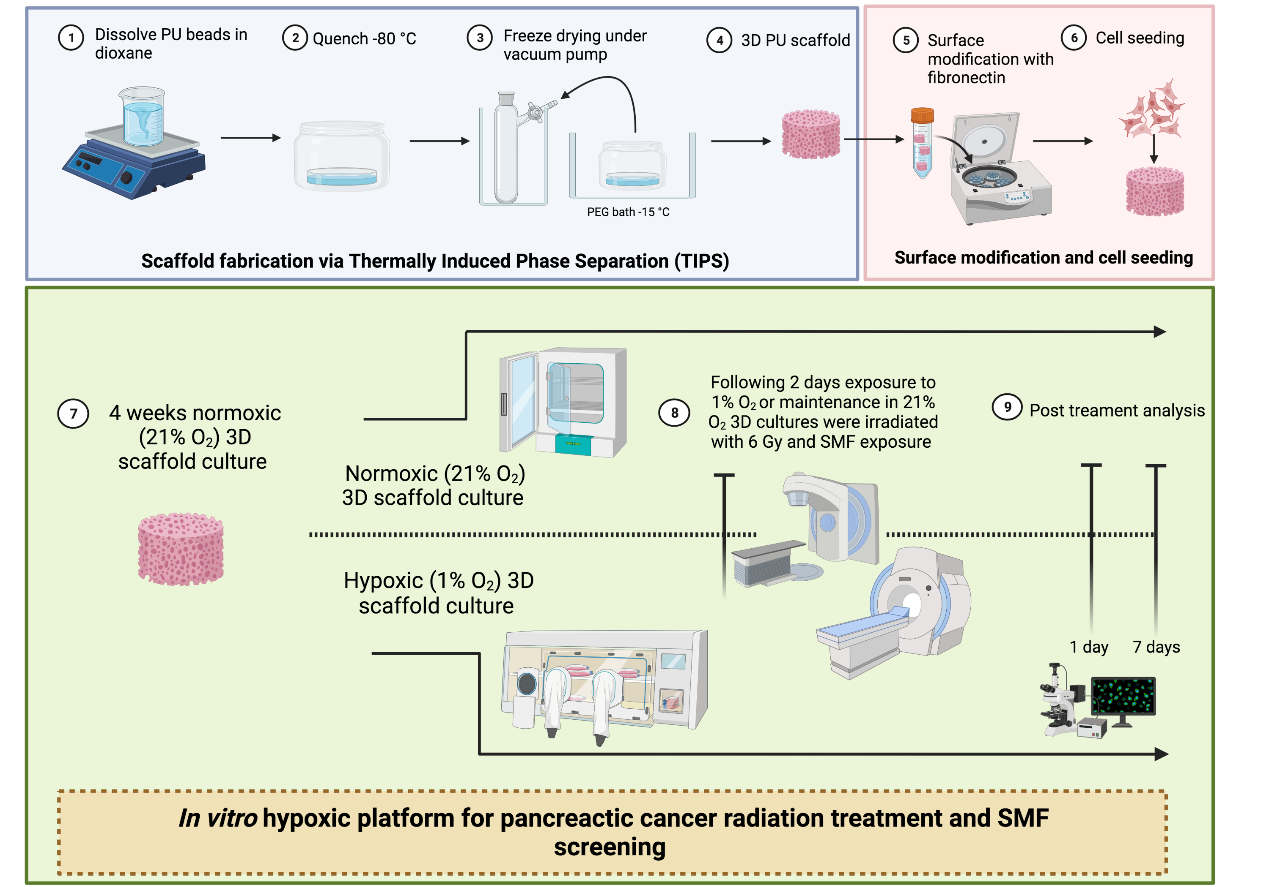
**

**Figure A1.** Experimental design. Including scaffold fabrication, surface modification and cell seeding. Created with BioRender.com
